# A narrative review of gaps in the provision of integrated care for noncommunicable diseases in India

**DOI:** 10.1186/s40985-020-00128-3

**Published:** 2020-05-13

**Authors:** Manoj Kumar Pati, N. Swaroop, Arin Kar, Preeti Aggarwal, Krishnamurthy Jayanna, Wim Van Damme

**Affiliations:** 1 Karnataka Health Promotion Trust, IT Park, 5th floor, 1-4, Rajajinagar Industrial Area behind KSSIDC Admin Office, Rajajinagar, Bangalore, Karnataka 560044 India; 2 Social Initiatives, Landmark Group, Bangalore, India; 3 Centre for Global Public Health, University of Manitoba, Winnipeg, Canada; 4 Health Policy Department, Institute of Tropical Medicine, Antwerp, Belgium

**Keywords:** Noncommunicable diseases, Health service delivery, Continuum of care, Urban health, Screening for NCDs, Follow-up and referral, Private health sector, Monitoring systems

## Abstract

**Background:**

Low- and middle-income countries (LMICs) account for a higher burden of noncommunicable diseases (NCD) and home to a higher number of premature deaths (before age 70) from NCDs. NCDs have become an integral part of the global development agenda; hence, the scope of action on NCDs extends beyond just the health-related sustainable development goal (SDG 3). However, the organization and integration of NCD-related health services have faced several gaps in the LMIC regions such as India. Although the national NCD programme of India has been in operation for a decade, challenges remain in the integration of NCD services at primary care. In this paper, we have analysed existing gaps in the organization and integration of NCD services at primary care and suggested plausible solutions that exist.

**Method:**

The identification of gaps is based out of a review of peer-reviewed articles, reports on national and global guidelines/protocols. The gaps are organized and narrated at four levels such as community, facility, health system, health policy and research, as per the WHO Innovative Care for Chronic Conditions framework (WHO ICCC).

**Result:**

The review found that challenges in the identification of eligible beneficiaries, shortage and poor capacity of frontline health workers, poor functioning of community groups and poor community knowledge on NCD risk factors were key gaps at the community level. Challenges at facility level such as poor facility infrastructure, lack of provider knowledge on standards of NCD care and below par quality of care led to poor management of NCDs. At the health system level, we found, organization of care, programme management and monitoring systems were not geared up to address NCDs. Multi-sectoral collaboration and coordination were proposed at the policy level to tackle NCDs; however, gaps remained in implementation of such policies. Limited research on the effect of health promotion, prevention and, in particular, non-medical interventions on NCDs was found as a key gap at the research level.

**Conclusion:**

This paper reinforces the need for an integrated comprehensive model of NCD care especially at primary health care level to address the growing burden of these diseases. This overarching review is quite relevant and useful in organizing NCD care in Indian and similar LMIC settings.

## Background

Four major noncommunicable diseases (NCDs) such as cardiovascular diseases, cancer, diabetes and chronic respiratory diseases are the leading global causes of death and are responsible for 70% of deaths worldwide in 2015 [1]. Three fourths of all these NCD deaths occurred in lower- and middle-income countries (LMICs) [2]. Recent reports on high-level meetings of United Nations General Assembly (UNGA) on NCDs [3, 4] suggest that an equitable and affordable primary care prevention, treatment and support system for NCDs is required to achieve universal health coverage (UHC). NCD targets cut across almost all sustainable development goals (SDG) [5]; hence, all high-burden countries should urgently prioritize actions towards this.

NCDs contribute to around 5.87 million deaths in India, accounting for 60% of all deaths in the country [1], and more than two-thirds of all NCD-related deaths in the entire South-East Asia Region (SEAR) [2]. The risk of an Indian dying between 30 and 70 years of age (premature deaths) from any one of the four major NCDs was about 23% in 2016 [6]. India is struggling to tackle the rising burden of NCDs due to epidemiological and demographic transitions. In such settings, the importance of primary health level care for NCDs cannot be overemphasized. In India, a majority (64%) of healthcare payments is through out-of-pocket expenditure [7]. Evidence shows that the burden of NCDs and the prevalence of related risk factors are relatively higher in urban areas of India [8, 9]. The prevalence is on the rise among the poor, making them particularly vulnerable to catastrophic health expenditure, in addition to life-long morbidity and increased mortality [10]. Further, many NCDs goes unreported due to lack of clinical symptoms and are deeply related to individual lifestyle and habits. Hence, primary healthcare is the preferred choice to focus on health prevention and facilitate early screening. Additionally, evidence suggests primary care is the best avenue for delivering NCD care in the most integrated and comprehensive way [11–13].

The government of India launched the national NCD programme called the National Programme for Prevention and Control of Cancer, Diabetes, Cardiovascular Diseases & Stroke (NPCDCS) in 2008. This national programme merged into the flagship National Health Mission (NHM) in 2013 aiming to integrate NCD-related programme activities related to health promotion, early diagnosis, treatment and referral and further facilitate partnerships with the private sector [14]. Despite such efforts and initiatives by the government, currently, there is no effective model which can demonstrate the integration of NCD-related services. It was apparent that the implementation approach followed in the national NCD programme was more suitable to the rural health system than an urban one until recently (2017). Very recently the programme recognized the importance of population-level screening and strengthening capacity-building efforts for primary care health care team and frontline health workers (FLWs) as crucial first steps to its aim of integration of services for NCD care [15]. Challenges remain in programme implementation plans; while the current government guidelines indicate sensitization of programme managers in service integration for effective management of NCDs [16], it lacks a proper plan of action. Similarly, there is a lack of comprehensive information pool and management information system to efficiently monitor the reach and implementation progress of the national NCD programme.

India was the first country to set national targets on NCDs in line with the global NCD action plan [17] and was among the first few to ratify and implement the WHO’s Framework Convention on Tobacco Control; yet such a high burden of NCDs demonstrates the challenges in the implementation of national guidelines at state and district level. Integrated service delivery models especially centred around primary care in urban areas are need of the hour to address the growing burden of NCDs [18]. Identifying gaps and solutions at different levels of intervention, integrating them into a package of interventions that is simple to implement and manage and building an efficient monitoring and evaluation system [19] are important for the development of sustainable NCD care models. Strengthening primary care for the integration of NCD services can be the beginning, and this paper highlights various gaps that hinder any such integration. Based on the gaps identified, we suggest an integrated NCD care model for primary care following an adapted version of the World Health Organization’s Innovative Care for Chronic Conditions (ICCC) framework. The suggested model could be useful in organizing NCD care in India and similar settings.

## Methods

### Review process

Through a narrative review of the literature, we used the WHO ICCC framework as a theoretical framework of knowledge to identify and organize gaps under four major themes—at the community, facility, health system and gaps at the health policy and research. We adapted sub-themes and components of the ICCC framework to the local context (India and Karnataka).

### Hypothesis

We hypothesize gaps at community, facility, health system and policy level hampers effective integration and continuity of care for NCDs.

We have contextualize ‘Integration of NCD Services’ by following definitions [20, 21]:

1. Integrating NCD services into primary health care
2. Integrating NCD services from a health systems lens: Integration of all interventions at community level (mostly towards disease prevention and promotion, early identification and enrol to care), facility (towards disease management and follow-up) and overall systems level (towards supplies of medicine and logistics, programme management, monitoring systems) and finally at the level of policy and research.
3. Integration of NCD services through a unified management approach: Integration of cross-cutting components like health promotion, prevention, screening of population, training, referral services, emergency medical services, public awareness programme management, monitoring and evaluation

Similarly, for the purpose of this study, we have defined ‘Continuity in Care’ more in terms of ‘management continuity’ and ‘informational continuity’ rather than ‘relational continuity (therapeutic relationship between a patient and one or more providers)’ [22]. We have used an established ‘continuum of care’ framework [23] as initial theoretical framework to focus on management continuity (i.e. starting from early screening, through confirmation and prompt treatment, patient follow-ups, to referral and complication management) and informational continuity (monitoring systems to track individualized treatment goals).

### Inclusion criteria

We included all articles and reports published after the year 2000 in the review and used information from peer-reviewed articles, reports, guidelines and policy documents to identify the gaps.

### Search criteria and search items

**Fig. 1:**
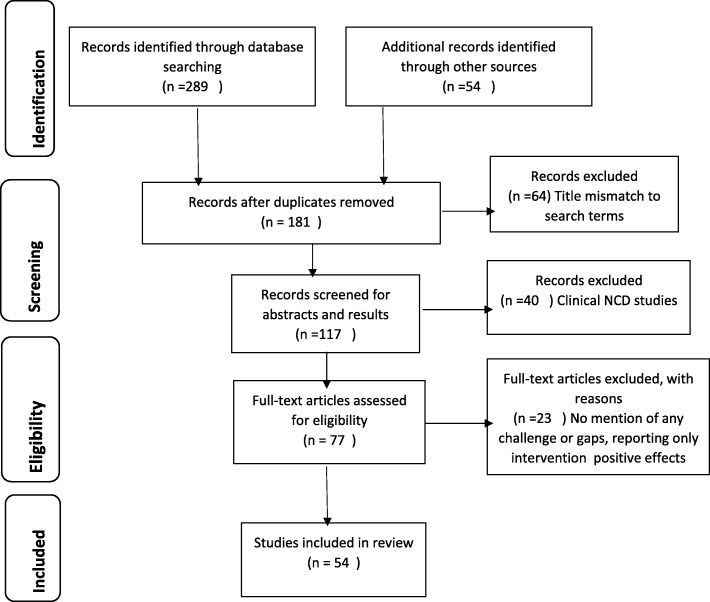
Flow chart for review of the literature (as per PRISMA 2009 guideline)

We conducted the literature review using MEDLINE (PubMed Central) and Google Scholar as search engines. Common search terms were ‘chronic disease care’, ‘primary care’, ‘NCD in primary care’, ‘NCD and urban health’, ‘early screening of NCDs in community’, ‘health system challenges in NCD care’, ‘programme management and NCD’, ‘NCD and policy’ and ‘India’ as an adjunct search item. The initial literature review yielded some 343 articles (including reports) of which some 117 were initially screened for abstracts and results, and 77 full-length articles and reports were included in the final review. Finally, we reviewed 54 documents including full-length peer-reviewed articles, reports and policy documents (Fig. 1).

## Results

### Gaps at the level of community

#### Population enumeration to cover the eligible population

Evidence shows that in India, geographical boundaries, especially those in urban areas, are often blurred. Revenue boundaries, judiciary boundaries and health services-related boundaries often do not match [24–26]. Additionally, a high level of in- and out-migration in urban areas pose a challenge to the enumeration of households [27, 28] making it difficult for resource allocation and organization of care. The planning of developmental programmes, including health service interventions, often suffers at the operational stage of defining the target population. Outreach activities including early screening and regular follow-up are impeded by interplay of a variety of factors such as blurred boundary, capacity constraint of the community health workers and lack of continuous supportive supervision.

#### The knowledge gap in the community on risk factors

Although the awareness of diabetes is on the rise, especially in urban areas, the understanding of risks remains poor [29]. A study in India found that 25% of the population was unaware of diabetes and only 22.2% of the population and 41% of known diabetic subjects felt that diabetes could be prevented [30]. Even among educated population comprising mainly postgraduates, medical professionals and lawyers, only 42.6% knew that diabetes could be preventable. The knowledge of risk factors of diabetes was even lower, with only 11.9% of study subjects reporting obesity and physical inactivity as the risk factors; 23% knew that diabetes could lead to foot problems; and only 5.8% knew it could cause heart attack [30]. There is a lack of standard protocol on diabetes education and self-management in the community leading to noncompliant with health seeking and intake of medicine [31]. In another study, among diabetics, 41.4% had not visited their health care provider in the last year; only 13%, 16.2%, 32.1% and 3.1% of respondents had undergone HbA1c test, eye examination, serum cholesterol test and foot examination, respectively [31]. Studies show that if risk factor targets were achieved, premature mortality from NCDs would decrease by 22% in men and 19% in women [32]; however, awareness does not necessarily always result in behavioural changes [33, 34]. However, few efforts have been made to address this knowledge gap on risk factors and improve health-seeking behaviour for NCDs in India [35, 36]. This has resulted in major hurdles for effective management of NCDs, especially in vulnerable geographies.

#### Gaps at the level of frontline health worker: shortage of manpower and lack of training

Another challenge to the early screening of NCDs in India is an inadequate number of trained FLWs to conduct population-level screening, health promotion and prevention activities as envisaged by the national NCD programme. As per government norm, each accredited social health activist (ASHA) is allocated per 1000 population in rural area but in case of urban areas this cadre is only allocated for 2500 in vulnerable and marginalized communities. In addition to this challenge, there is frequent vacancy of ASHA posts in urban areas given better availability of other job opportunities. Although recent government guidelines have delineated the role of FLWs such as auxiliary nurse midwives (ANMs) and ASHAs in early screening, referral and follow-up [14] of NCDs, FLWs across facilities in the country are yet to be fully sensitized on NCD care. For example, recent guidelines prescribe a tool for ASHAs, called the ‘Community-Based Assessment Checklist’ for early risk identification of common NCDs; however, the training is still ongoing and it is yet to be implemented in the field [15]. ASHAs lack handholding support in the community and are visibly overburdened by the number of tasks they are expected to perform; they are at a time expected to perform the role of an educator, link worker, service provider and activist which affect their performance [37–39]. ANMs are very recently trained on early screening of NCDs and care thereafter; however, the population screening programme is yet to be fully rolled out. Currently, the role of these frontline workers has been largely limited to carrying out maternal and child health interventions [40, 41].

#### Limited role of community groups in NCD care

Community groups such as Village Health Sanitation and Nutrition Committee (VHSNC) and MAS (Mahila Arogya Samiti^1^) are not well acquainted with the NCD programme and lack training to focus on the integration of NCDs into existing community monitoring mechanisms. Often MAS are non-functional in the urban areas making it difficult to sensitize them on NCD care.

In summary, community-level gaps pose a major challenge to early screening for NCDs and linking patients to care in time.

### Gaps at the level of a healthcare facility

#### Gaps in the referral and follow-up of NCD cases

Effective management of NCDs starts with effective referral system [20, 21] which include referral of suspected cases for confirmatory tests, referral of patients to higher facilities for complication care and referral back to the community to maintain the continuity of care. Referral and follow-up of suspected cases to the nearest public (or private) facility for confirmation should follow post-screening of NCDs. At this stage, FLWs encounter several challenges. Lack of standardized referral protocols, referral forms and inadequate training of FLWs in the public health system are often the reason behind delay to the confirmation process. As a result, many patients either visit private facilities leading to out-of-pocket expense on confirmation tests or do not visit any facility leading to treatment delay. Those who do reach facilities are often not adequately oriented and counselled resulting in unnecessary anxiety about the diagnosis. These difficulties affect the continuity of care. NCD clinics are established at the secondary level or higher levels to facilitate care and treatment for referred cases as part of the national NCD programme [14]. However, the effectiveness of such clinics in delivering comprehensive care remains unknown. In summary, a weak referral and care system defeats the purpose of early screening.

#### Gaps in facility readiness and provider capability

Adequate facility readiness and provider preparedness are prerequisites for early initiation of treatment and high quality of NCD care. However, the public health system in India, which is supposed to be the principal custodian of people’s health, is faced with multiple challenges at every level, mainly related to the readiness of facilities in terms of infrastructure and the preparedness of providers in terms of delivering standardized NCDs care [42–46]. Delayed diagnosis of diabetes and hypertension has been reported by many studies in India. In a Pan-India study, only 43.4% patients had their BP checked at the time of diagnosis; the figures were 17.6%, 5.6% and 4.2% for eye examination, kidney function tests and lipid tests, respectively, for diabetics with complications [47]. At the facility level, there is a lack of timely supply of drugs and medical equipment including diagnostic kits, lack of trained providers such as medical officers, pharmacists and laboratory technicians [47]. A study found that, only 10–12% of people with diabetes received modern pharmacological treatment in India on an average. The availability of key oral anti-diabetic drugs such as Glibenclamide varied from 100% in the state of Karnataka to 3.8% in West Bengal. Although insulin therapy is accepted as one of the most effective and dependable treatment option, barriers to its use were identified [47]; In most patients, insulin was delayed until it was absolutely necessary or when the HbA1c levels had deteriorated further to approximately 9%. Inadequate use of patient medical records and improper adherence to standard treatment protocols affect clinical decision-making; only six of the 15 health facilities in an urban health study had a system that tracked medical records of diabetes patients and providers in all these facilities expressed that there were no such thing called standard treatment protocols and treatment depends upon individual analysis of patient condition [48]. This results in incomplete and irregular treatment leading to complications which ultimately bring economic and social liability along with suffering [49]. Although standard diagnosis and treatment protocols for NCDs are available, providers are yet to be trained on those protocols. Counselling and follow-up services also suffer in public health facilities due to lack of trained counsellors. For instance, absence of trained diabetic educators leaves the onus of counselling and educating patients on already overburdened physicians, and lack of standard counselling messages further result in inconsistent patient education and adherence to treatment [50]. Evidence shows that there is an age shift in the onset of NCDs in India, from an adult population group to relatively younger groups [51]. With the early onset, there is ample time for NCDs to develop complications given awareness is poor and the health system is not yet prepared to tackle the growing burden [52]. Systematic referral linkages have not yet been established across facilities for NCD treatment and complication management. Complications from NCDs lead to an increasing number of premature deaths [53] causing increased financial burdens on families and the nation as a whole. Citing delayed diagnosis, sub-optimal glycemic control and failure to screen for early-stage complications as three major reasons for these deaths, the study reported that 47% of the diabetes cases in the population were undiagnosed highlighting the poor awareness and detection of diabetes in India. It is important to note that 43% of deaths due to diabetes in India in 2016 were in people younger than 70 years again indicating possible inadequacies in diabetes management [53].

#### Increased reliance on the private health sector for NCD care despite unknown quality of care

Given the challenges associated with the public health system, the private health sector remains the preferred choice of care in India. Private practitioners, both qualified and unqualified, play a major role in health care delivery in India despite concerns about the practice of inconsistent treatment protocols, unnecessary diagnostic tests and high treatment costs [54]. The private sector now accounts for 60–80% of all out-patient health care in India [55] which leads to high health expenditures [48, 56–58]. This situation is similar for NCDs as many patients regularly access private health care of inconsistent and non-uniform quality.

In summary, facility-level gaps pose a challenge to the effective management of NCDs and related complications.

### Gaps in the public health system, programme management and monitoring systems

India’s public health system is not aligned and prepared to tackle chronic diseases posing a major hurdle to the organization of care and referral services for NCDs [59–61]. Current health systems are not able to plan for population-level NCD interventions so far due to the scale of the problem. More focus is being put on curative care and the setting up of NCD clinics, rather than development and implementation of an integrated model. Sub-optimal utilization of existing resources, below par programme management techniques and lack of planning, has affected the implementation of the well-designed chronic care programme [62]. Further, the programme managers often lack capabilities in design, planning, implementation, monitoring and evaluation of NCD programmes due to inadequate training modules, process and tools, lack of data systems and underuse of technology [63].

### Gaps at policy and research level

For effective delivery of NCD care at primary care level, attention and actions from policymakers on strategic planning [64] are required. Different types of NCDs are grouped within the current national programme, yet it may not make sense to approach them with a common strategy in the field; for example, the care approach to hypertension and to that of cancers should be very different from each other. This makes the delivery of primary care NCD services labour intensive and challenging. For example, the existing health system is not yet ready to handle an increasing burden of mental health illnesses in India as envisaged in the national NCD programme [65].

Further to this, the effective prevention, screening and treatment of NCDs require action and coordination from non-health stakeholders such as academia, legislation, urban planning, environment and food industry. Although indicated in policy documents such as the 5-year plan [66], progress in intersectoral coordination has remained limited. There are implementation gaps for interventions (promote physical activity in schools and society, restrict marketing of and access to food products high in salt, sugar or unhealthy fats) and legislations (clean indoor air legislation, tobacco advertising ban, raising tax on tobacco products) necessitating intersectoral coordination.

Another area that warrants attention is the need to expand investments in the area of implementation research around the prevention and promotion of NCDs. Currently, research and programme evaluations are skewed towards therapeutic/curative side. Research in understanding the role of alternative systems of medicine in health promotion and disease prevention is limited. Considering large vacancies of trained counsellors at urban primary care, role of counsellor in primary and secondary prevention of NCDs could be studied.

**Fig. 2:**
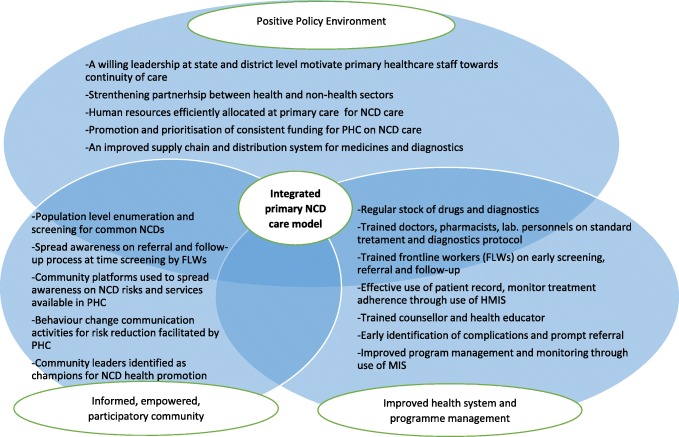
Integrated primary NCD care model adapted from the WHO Innovative Care for Chronic Conditions (ICCC) Framework

Based on the gaps identified in this article, we developed an integrated NCD care model for primary care based on an adapted version of the WHO ICCC framework (see Fig. 2). The WHO framework informs health systems to update its health care to meet the needs of chronic conditions. The building blocks of this framework are relevant for both prevention and disease management in health care settings. We are currently implementing this integrated NCD care model as a pilot intervention in one of the urban primary health centre catchment area in Mysore city of Karnataka state in India [18].

## Conclusion

This review identified gaps in NCD care in India at various levels such as identifying target population, early screening through disease management to programme management and policy level. Given the large and versatile geography of Indian subcontinent, NCD burden and response widely varies across states; while the burden is relatively higher in comparatively richer southern and western states, response and infrastructure in these states are also relatively better than northern and eastern counterparts. Similarly, rural and urban differences are huge in term of NCD prevalence; urban dynamics are different with large scale migration, presence of a pluralistic health system and complex patient pathways. However, barring few exceptions, we did not find any major differences across states or urban-rural differences for the majority of gaps identified. We have analysed the presence and distribution of such gaps across state and rural-urban regions through an additional excel file (Additional file 1); we found that majority of gaps holds good for most of the Indian states and across rural and urban geographies. There were few exceptions though such as enumerating the target population was found to be a major gap in urban areas compared to rural due to poorly defined geographical boundaries and continuous large scale migration. Poor community awareness on NCD risks and prevention found to be more in poor, remote and vulnerable geographies across states and urban-rural settlements. Overall patient awareness on disease management and self-care for NCDs found to be poor in both rural and urban geographies. While, northern and northeastern states found to be lagging behind in training frontline cadres on NCDs, in southern states, availability per se of such cadres was a challenge especially in urban areas. Facility-level disease management gaps existed more or less similar across states and urban-rural areas. Although, health system response and policy dialogues for NCDs were in a little progressive stage in southern states; we did not find any major differences in action towards NCDs among different states. While the public facilities are not geared up for addressing growing burden of NCDs, private health care for NCDs comes with very high cost and of questionable standards.

Overall, there is a lack of prioritization of NCD services at the primary healthcare level in India, such that people with NCDs in India are highly reliant on the private health sector where the quality and standard of care is uncertain and where the government has no control on overpricing. There is a lack of knowledge among people about available NCD services in the public health system and little attempt is made to design interventions to target high-risk and vulnerable communities. Frontline health workers lack training in standards of referral and follow-up for presumptive, newly diagnosed and existing cases of NCDs. Although standard referral and follow-up protocols are available, these are far from being effectively institutionalized and implemented in its entirety. Gaps in programme management lie in identifying the most cost-effective local interventions for NCD care and in setting up monitoring and evaluation systems for NCD programme at primary care level. Although training manuals are available for programme managers, programme managers across India are yet to be trained in designing and implementing comprehensive NCD care programmes.

It is thus very important for the NCD programme to expand from the current ‘screening focused’ approach at primary healthcare level to an integrated approach of ‘community engagement’ for health promotion, risk reduction and ‘provider and facility readiness’ for effective treatment, management and rehabilitation. Learnings and strategies from existing chronic disease care programme could be adapted such as the innovative Detect-Treat-Prevent-Build strategy being used in the Revised National Tuberculosis Control Programme (RNTCP) [67]. Preventing through customized behaviour change communication for common risk factors and individual patient counselling for adhering to treatment and follow-up, detecting through systematic screening of target groups (age group, tobacco use, obesity etc.) and involvement of private providers in detecting NCD cases could be adapted. Similarly, treating through a trained multi-disciplinary team and through use of fixed dose combination of anti-diabetic or anti-hypertensive drugs [68] just like TB drugs could be practiced at primary care settings. Finally, building a supportive system is of much importance; continued supply of medicines, adequate staffing, continuous capacity building of primary care team on NCD care competencies, supportive supervision and mentoring and a strong data monitoring system could all be adapted from the TB programme. However, instead of a vertical approach as with RNTCP, the DTPB strategies could be adapted for NCDs within existing PHC structure.

Thus, ***integrating NCDs at primary health care helps in managing NCDs at an early stage and therefore is a better investment than diagnosing and managing them at a later stage*** [20]. Enumeration and tracking of eligible populations need to be strengthened [69] to aid the initiation of early screening and provision of effective care [70]. There is an urgent need to build the capacity of existing health workers on early identification of disease and risk factors using standardized tools. The effective screening will help in detecting the early onset of NCDs as well as tracing the high-risk groups in the community [18, 71]. It is also critical to engage with the community and encourage their active participation in the NCD programme; behaviour change communication (BCC) activities focusing on NCD risks and prevention strategies [72] are vital. Systematic assessments of facility readiness and provider capability for the provision of NCD services are urgently needed. Assessments can be adapted from other programmes like MNCH programme [73], and standardized checklists and treatment protocols can be used. Guidelines can be framed to leverage the potential of key private healthcare facilities in streamlining referral and follow-up to ensure the provision of standardized care at an affordable price.

Similarly, programme monitoring and evaluation are important for strategic planning; it is timely to revisit monitoring indicators of the national NCD programme and strive for a sustainable surveillance system [19]. Understanding the burden of disease through population-level data systems is essential; evidence suggests that development of monitoring indicators and an information system are vital for effective programmatic decision-making under the NCD programme in India [24, 38]. Lessons and good practices can be adapted from other programmes such as the National AIDS control programme, where a national web-enabled strategic management information systems improved programme management and monitoring situation for AIDS control in India [74].

An understanding of the patient landscape and specific global models can inform technological policy innovations which can lower barriers to effective NCD screening, diagnosis and management [75]. The WHO Package of Essential NCD Interventions (WHO PEN) is such an action-oriented set of cost-effective integrated interventions at the primary health care level and is documented to be the most effective in tackling health system issues [20]. Integration could be happened across different national health programme as well to optimize health system resources, e.g. breast cancer and cervical cancer, which have gender implications, could be better screened and handled if integrated into RMNCH+A programme [76]. A policy can be chalked out to encourage women from participating in community breast and cancer screening programme [77]. India should acknowledge the growing burden of mental health disorders and could set an example by [78, 79] including mental health in its national NCD programme. Policies to create a positive environment for promoting healthy lifestyles need to be prioritized. Also, the government should consider the impact on health while planning and implementing other non-health-related policies. Harnessing interventions from AYUSH, such as yoga, breathing exercises, meditation and others, and evaluating them through rigorous scientific methods to understand their efficacy in NCD prevention and health promotion is important.

## Supplementary information

**Additional file 1:.** Mapping of reviewed studies across identified gaps. All reviewed studies presented in the result section are mapped for study locations (states and urban-rural location) across identified gaps

## Data Availability

Not applicable

## Abbreviations

ANM: Auxiliary nurse midwife
ASHA: Accredited social health activist
DTPB: Detection, Treatment, Prevention, and Build
LMIC: Low- and middle-income countries
MAS: Mahila Arogya Samiti
NCDs: Noncommunicable diseases
PHC: Primary health centre
RMNCH+A: Reproductive, Maternal, Newborn Child and Adolescent Health Programme
RNTCP: Revised National Tuberculosis Control Program
SDG: Sustainable Development Goals
SEAR: South-East Asia Region
UNGA: United Nations General Assembly

## Footnotes

1 MAS is one of the key interventions under National Health Mission aimed at promoting community participation in health at all levels, including planning, implementing and monitoring of health programmes. MAS is expected to take collective action on issues related to health, nutrition, water, sanitation and social determinants at the slum level. It is envisaged as being central to ‘local collective action’, which would gradually develop to the process of decentralized health planning. Each MAS covers around 50–100 households in slums or slum-like settlement, and function as a local women’s collective with an elected chairperson or secretary while ASHA facilitates as member secretary.
